# Local Growth Hormone Therapy for Pressure Ulcer Healing on a Human Skin Mouse Model

**DOI:** 10.3390/ijms20174157

**Published:** 2019-08-26

**Authors:** Lara Cristóbal, Nerea de los Reyes, Miguel A. Ortega, Melchor Álvarez-Mon, Natalio García-Honduvilla, Julia Buján, Andrés A. Maldonado

**Affiliations:** 1 Department of Plastic and Reconstructive Surgery and Burn Unit. University Hospital of Getafe, 28905 Madrid, Spain; 2 Department of Medicine and Medical Specialties, Faculty of Medicine and Health Sciences, University of Alcalá, 28801 Madrid, Spain; 3 Ramón y Cajal Institute of Sanitary Research (IRYCIS), 28801 Madrid, Spain; 4 Networking Biomedical Research Center on Bioengineering, Biomaterials and Nanomedicine (CIBER-BBN), 28801 Madrid, Spain; 5 Immune System Diseases-Rheumatology and Oncology Service and Internal Medicine Department, University Hospital Príncipe de Asturias, Alcalá de Henares, 28805 Madrid, Spain; 6 University Center of Defense of Madrid (CUD-ACD), 28047 Madrid, Spain; 7 Department of Plastic, Hand and Reconstructive Surgery, BG Unfallklinik Frankfurt, 60389 Frankfurt am Main, Germany

**Keywords:** growth hormone, human skin graft, pressure ulcer treatment, wound healing

## Abstract

The growth hormone is involved in skin homeostasis and wound healing. We hypothesize whether it is possible to improve pressure ulcer (PU) healing by locally applying the recombinant human growth hormone (rhGH) in a human skin mouse model. Non-obese diabetic/severe combined immunodeficient mice (*n* = 10) were engrafted with a full-thickness human skin graft. After 60 days with stable grafts, human skin underwent three cycles of ischemia-reperfusion with a compression device to create a PU. Mice were classified into two groups: rhGH treatment group (*n* = 5) and control group (*n* = 5). In the rhGH group for local intradermal injections, each had 0.15 mg (0.5IU) applied to the PU edges, once per week for four weeks. Evaluation of the wound healing was conducted with photographic and visual assessments, and histological analysis was performed after complete wound healing. The results showed a healing rate twice as fast in the rhGH group compared to the control group (1.25 ± 0.33 mm2/day versus 0.61 ± 0.27 mm2/day; *p*-value < 0.05), with a faster healing rate during the first 30 days. The rhGH group showed thicker skin (1953 ± 457 µm versus 1060 ± 208 µm; *p*-value < 0.05) in the repaired area, with a significant decrease in collagen type I/III ratio at wound closure (62 days, range 60–70). Local administration of the rhGH accelerates PU healing in our model. The rhGH may have a clinical use in pressure ulcer treatment.

## 1. Introduction

Despite the implementation of prevention strategies, the incidence and prevalence of pressure ulcers (PU) in today’s society is high. Because of population aging and medical advances, this disease has become a health problem, which also entails social and economic impact. [1] Research on the pathogenesis and mechanisms involved in PU and wound healing is crucial. However, research in this field has been hindered by a lack of experimental animal models [2].

Regarding the etiology of PUs, external pressure is viewed as the main factor. Other patient-specific factors leading to derangement in tissue perfusion may account for an observed development of a pressure ulcer. It is well known that ischemia–reperfusion injury contributes to the pathophysiology of PUs more significantly than a single, prolonged ischemic insult [3,4]. Previously published PU models have been developed using animal skin, which imposes limitations when studying the wound healing process and the extrapolation of results and the effect of treatments on humans. 

Once a pressure ulcer has developed, its course becomes insidious and its evolution towards healing is prolonged. The management of these patients needs a comprehensive and multidisciplinary team approach, involving plastic surgeons, nurses, nutritionists, rehabilitation therapists, etc. The goal in these chronic wounds is prompt tissue recovery. [1,5] Treatments that accelerate wound closure are of vital importance and interest for its potential benefits for these patients. The available evidence supports the use of different therapeutic options based on patient and wound characteristics, but none preponderates as the gold standard. Tissue engineering applied to wound healing, and specifically to PU, and approaches incorporating cellular therapy and growth factors are an expanding field in this issue. According to the American and European Pressure Ulcer Advisory Panel guidelines, Platelet-Derived Growth Factor (PDGF) can be used for Category/Stage III and IV pressure ulcers that have delayed healing. However, the evidence is not sufficient to recommend or refute the routine use of other growth factors in the treatment of pressure ulcers. [6] Rees et al. [7] published a randomized double blind, placebo-controlled study in which the efficacy of becaplermin gel (recombinant human PDFG-BB) in the treatment of chronic full thickness pressure ulcers was compared to that of placebo gel. The authors concluded that within the setting of a comprehensive wound management program, becaplermin gel once daily increases the incidence of complete healing and ≥90% healing in patients with full thickness pressure ulcers. Another randomized double blinded study [8] suggested that treatment with PDGF-BB before surgery enhances the ability to achieve a closed wound over surgery alone. 

The growth hormone (GH), anabolic hormone, regulates growth through hypertrophy, hyperplasia, and as a result of tissue differentiation, cell proliferation and protein synthesis. It can exert its effects directly on the tissues, or indirectly through mediators: the so-called insulin-like growth factors IGF-I and IGF-II. [9,10] GH activity, sometimes mediated via IGF-I, is involved in skin homeostasis and wound healing acting at different stages and including dermal–epidermal communication [11]. During the inflammatory phase of healing, macrophages deliver growth factors that attract fibroblasts and facilitate the proliferative phase. GH promotes the release of some of these factors, such as EGF, VEGF, and FGF. Furthermore, IGF-I and II mRNAs are modulated during the healing process. The highest levels of IGF-I are seen in human wound fluids within 24 h after injury, and they return to their baseline when healing ends. [12,13] Animal models reveal that systemic GH promotes cellular proliferation, especially fibroblasts; granulation tissue formation; increases collagen formation and extracellular matrix; and enhances keratinocyte migration, shortening the time for healing, as well as providing increased mechanical strength of the wounds. [14,15,16,17,18,19,20,21,22,23] GH is also an inducer of the immune system. It mainly acts on macrophages and T-lymphocytes, that also play a key role in wound healing. [24] Studies in burns show that rhGH treatment could result in improved wound healing and reduced length of hospital stay. [25] Most of the studies have been developed applying systemic GH, and adverse effects cannot be ignored. [26]

The aim of this study is to compare the healing rate and different histological parameters on a previously described PU human skin model with and without the application of the recombinant human growth hormone (rhGH). We hypothesize that the local administration of the rhGH should increase the healing rate of PU and improve the quality of the human skin. 

## 2. Results

Five mice received local treatment with the growth hormone (rhGH group) and five did not receive it (control group). Two paired mice (one of each group) died during the surgical procedure. Local administration of the rhGH was well tolerated. No local reactions or adverse effects were observed in the rhGH group.

### 2.1. Macroscopic Evaluation

**Figure 1:**
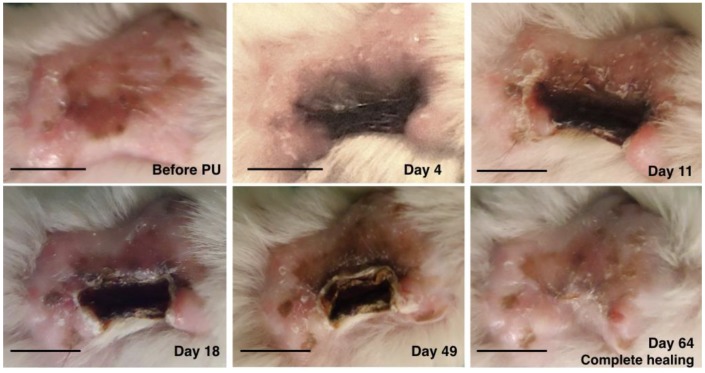
Macroscopic evolution of pressure ulcer healing in the recombinant human growth hormone rhGH group. The healing rate was 1.03 mm^2^ per day in this mouse. Bar = 10 mm.

**Figure 2:**
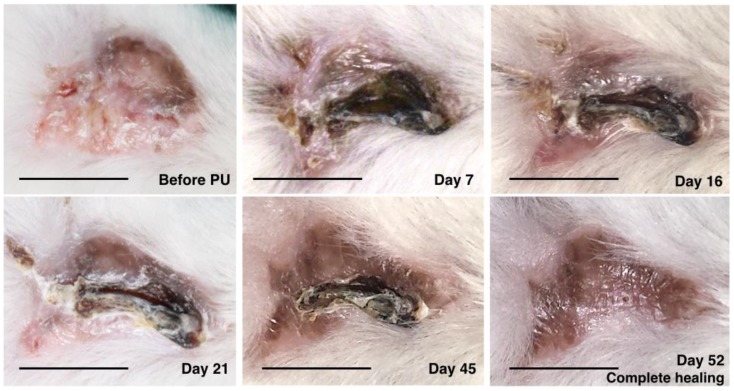
Macroscopic evolution of pressure ulcer healing in the control group. The healing rate was 0.75 mm^2^ per day in this mouse. Bar = 10 mm.

The healing pattern was similar among all mice. The macroscopic evolution of the PU was comparable between the rhGH group and the control group. The evolution was towards a retracted and elevated crusty lesion. This crust was giving off progressively from the periphery toward the center as the ulcer was healing. Fully recovered dermoepidermal tissue then emerged. The evolution of PU in the rhGH group and the control group can be observed in Figure 1 and Figure 2 respectively.

**Figure 3:**
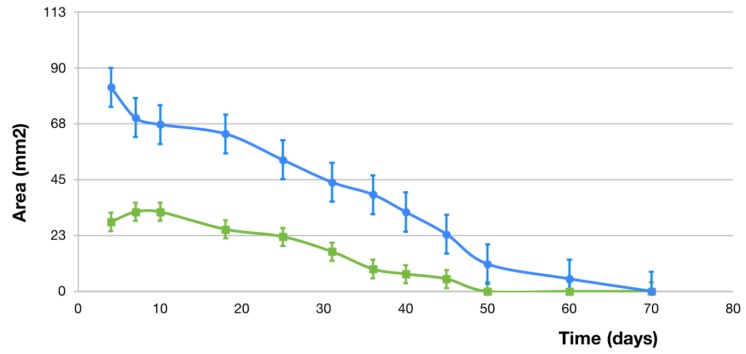
Daily pressure ulcer mean size (mm^2^) until complete healing in the rhGH group (blue line) and the control group (green line).

The healing rate was twice as fast in mice subjected to treatment with the growth hormone—rhGH group (1.25 ± 0.33 mm^2^ per day) compared to those which did not receive it—control group (0.61 ± 0.27 mm^2^ per day), with a statistically significant difference (*p*-value = 0.03). Kinetics was compared between groups, with faster healing for the first 30 days in the rhGH group (1.27 mm^2^ vs. 0.37 mm^2^), time in which the hormone was administered once per week. The daily PU mean size in both groups until complete healing can be observed in Figure 3.

**Figure 4:**
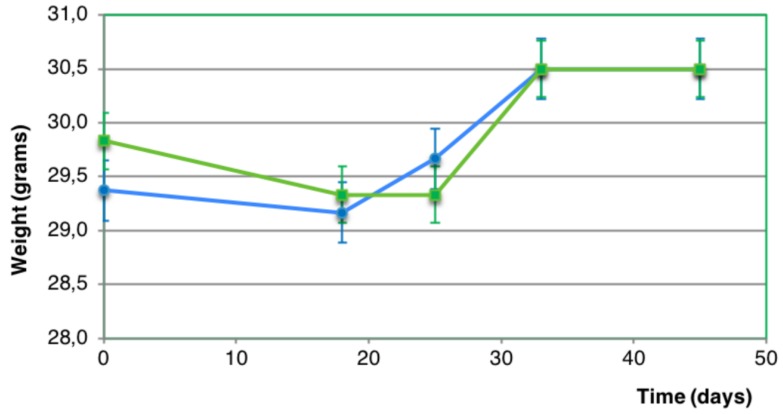
Evolution of body weight (grams) in the rhGH group (blue line) and the control group (green line). No statistically significant difference was found between both groups (*p*-value > 0.05).

Mean body weight in the rhGH group before PU formation and after PU healing was 29.38 ± 0.48 g and 30.2 ± 0.3 g respectively. Mean body weight in the control group before PU formation and after PU healing was 29.8 ± 1.9 g and 30.5 ± 2.1 g respectively. No statistically significant difference in body weight was found before and after PU healing in both groups (*p*-value > 0.05) (Figure 4).

### 2.2. Microscopic Evaluation

**Figure 5:**
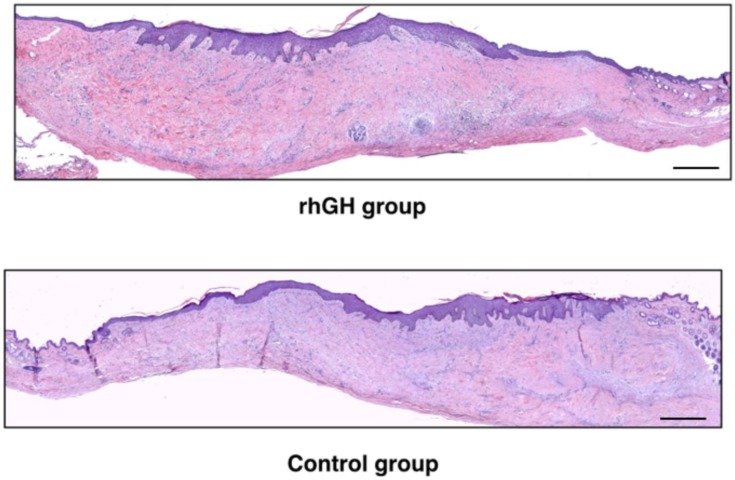
Histological analysis (H&E at 50 ×, panoramic view) of the rhGH group and the control group after complete healing of the pressure ulcer. Bar  =  200 µm.

**Figure 6:**
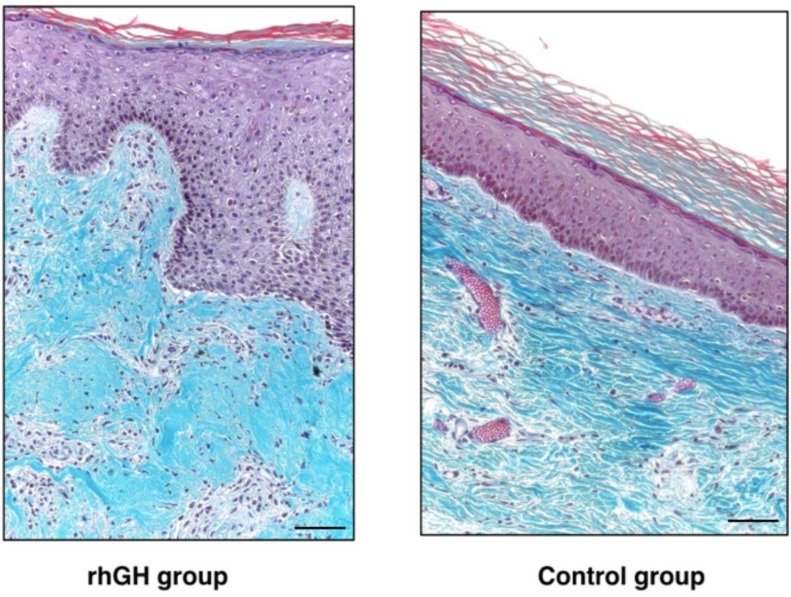
Histological analysis (Masson’s trichrome at 200 ×) of the rhGH group (left) and the control group (right) after complete healing of the pressure ulcer. Note the thicker dermis in the rhGH group. Bar  =  50 µm.

Epidermis maintained a normal architecture in both groups (Figure 5). No histological signs of malignancy were observed. The rhGH group showed thicker skin compared to the control group (1953 ± 457 µm versus 1060 ± 208 µm; *p*-value = 0.03) in the repaired area (Figure 6). This increase in the dermal layer was mainly due to a higher number of cells and the increase of extracellular matrix (rich in amorphous material). An increased in collagen deposition and a greater number of inflammatory cells were observed in the rhGH group.

**Figure 7:**
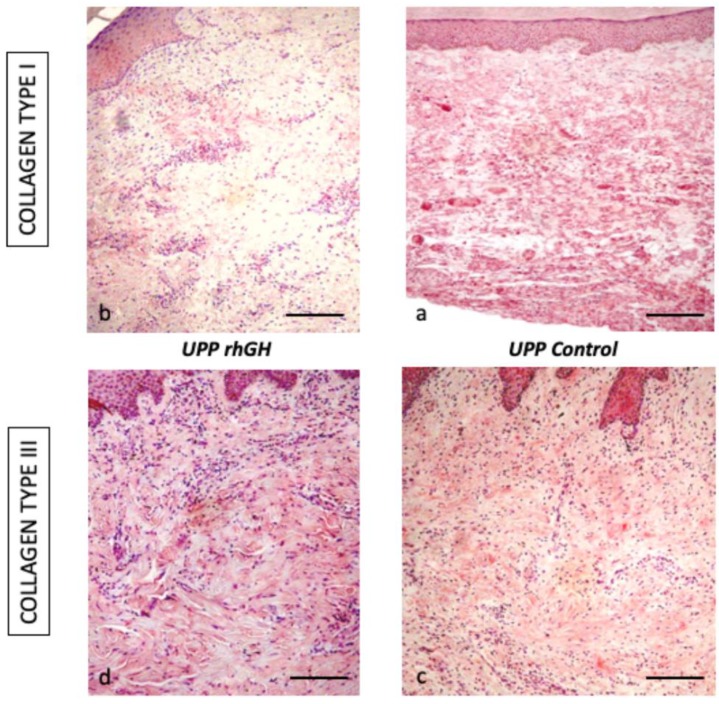
Immunohistochemical expression of collagen type I (160 ×) and III (250 ×) in the rhGH group (left, **a** and **c**) and the control group (right, **b** and **d**). Bar  =  50 µm.

The rhGH group presented an important decrease of collagen type I compared to the control group (16.8 % ± 4.4 versus 76.8 % ± 16.5; *p*-value < 0.01) and an increase in collagen type III (64.4 % ± 3.8 versus 70.8 % ± 2.8; *p*-value = 0.04) at wound closure (62 days, range 60–70) (Figure 7, Table 1).

## 3. Discussion

Wound therapies acting on a molecular and cellular level, such as growth factors, have a main role in wound healing. [27,28,29] The human GH, as an anabolic agent, stimulates growth and mitosis in a number of cell types by acting both directly and indirectly through the insulin-like growth factor (IGF)-I. Its effects have been proved in a variety of tissues, including skin, nerve, muscle, bone, cornea, etc. [11,14,17,19,22,23,30,31,32,33,34] and animal models reveal that systemic GH promotes granulation tissue and collagen formation, increases extracellular matrix, and enhances keratinocyte migration, shortening the time for healing. [14,17,19,22,23] Our findings show a statistically significant difference in healing rates between the rhGH group and control group, the healing rate being twice as fast in mice subjected to treatment with the rhGH. The faster healing was for the first 30 days in the rhGH group, time in which the hormone was administered once per week. 

In our histological analysis, we found a thicker skin, increased cellularity and decreased collagen type I in the dermis of the rhGH group. An increase in collagen type III with a decrease in collagen type I are indicators of a delay in the maturation period of collagen. All these characteristics are typical of an active prolongation of the dermal proliferation and secretion period (facilitating the sliding of the keratinocytes), and they should accelerate and improve the re-epithelialization process. This effect was observed in previous studies on diabetic rats with a polymeric GH delivery system. [18] Epidermis maintained a normal architecture and keratinocyte differentiation, showing no histological signs of malignancy. These data are consistent with the results published by Rudman et al. [35], Jorgensen et al. [36], and Conte et al. [37]. A recent study in mice published by Messias de Lima et al. [38], topical treatment with GH resulted in faster wound closure rates. The GH accelerates the closure of skin wounds by resolving the inflammatory phase faster, accelerating reepithelialization, collagen deposition, and stimulating angiogenesis.

The extracellular matrix (ECM) defines the mechanical properties of the skin. Collagens are a principal component, and type I and III collagens (fibrillar collagens) are the major determinants of the strength and stiffness of the tissue. [39] Both types of collagens have a very similar biochemical composition, and they are secreted as procollagens, containing a non-collagenous C-teminal propetide and a N-teminal propeptide. Propeptide processing may be complete in collagen I and incomplete in collagen III, leaving a C-telopeptide and a partially processed N-propeptide domain. These domains have been implicated in the regulation of fibrillogenesis [40]. In human skin, the ratio of collagen I/III is 1 for adolescent, increasing up to 2.5 in adult skin. This ratio depends on age, increasing in old age and decreasing in fetuses. The presence of a high content of collagen III could provide more flexibility and tensility. During wound healing the content of this type of collagens could modulate the scar formation. There is a direct relationship between a high secretion of collagen type I and hypertrophic scar formation. [41] Other authors have shown the importance of collagen type III in fibrinogenesis compare to collagen type I [42]. The authors mentioned how collagen type III was present in distensible organs, reducing the mechanical stiffness. The alteration of the ratio of both collagens can be indicative of a tissue response process to an adverse event, such as an abdominal hernia. [43] In situations of chronic stress and injury, tissues are able to react with a change in the ratio of collagens, increasing the gene and protein expression of collagen type III [44]. The ECM protein profiles and distributions were examined in our model. The injury caused by the compression device is slightly different from other types of damage with heavy bleeding. Our model presents a progressive ischemia and necrosis formation, with progressive tissue regeneration from the healthy edges, showing an increase in the protein expression of collagen I and III in the human skin [45]. 

It is well known that GH is one of those molecules with pleiotropic actions on skin cells, and it participates in inflammatory, proliferative, and maturation phases of wound healing. During the inflammatory phase, macrophages deliver growth factors that attract fibroblasts and facilitate the next phase. GH promotes the release of (a) EGF, responsible for stimulating fibroblasts; (b) VEGF, which promotes angiogenesis in the wound; (c) FGF, which stimulates macrophages, mast cells, and T-lymphocytes, and facilitates granulation and epithelization [12]. Angiogenesis plays a key role during the granulation phase and tissue remodeling, as new vessels are required for the progression of wound healing. Endothelial cells express the GH receptor (GHR) [46] and produce the participation of GH in the latter process. In our PU model, the local treatment of the PU with rhGH produced changes in the ratio of the protein’s expression (collagen type I/III). A significant increase of collagen type III protein expression was observed after wound closure (62 days, range 60–70). This finding could be interpreted as a delay in the process of consolidation of the scar tissue, keeping the immature tissue longer. However, we hypothesize that these changes increase the tissue effectiveness, favoring the response capacity to cover the damaged area. This fibrillar collagen could potentially be able to mature and generate a skin with greater tone. 

Studies in patients who present delayed wound healing and/or catabolic states, such as diabetes or burns, showed that systemic GH treatment improves skin healing and reduces time required for wound healing. These patients present catabolic responses, with negative nitrogen balance and reduction in serum levels of GH and IGF. [47,48,49,50] A review of randomized controlled trials showed that people with large burns could benefit from using systemic rhGH because of faster healing of the wound and donor site and reduced length of hospital stay, without increased mortality or scarring. However, it seems to be related to an increased risk of hyperglycemia. [25] Patients with PU are patients with multiple comorbidities. In the case of the elderly, especially in hospitals, they are usually malnourished or have catabolic states that worsen the tissue repair processes, resembling the situation of burn or diabetic patients. Therefore, the rhGH could potentially have an important role in accelerating the PU healing in this population. We think that our immunosuppressed model resembles the general condition that many patients with PU present in clinical practice. Since pressure ulcers are not just a problem of pressure, many of the patients are characterized by advanced age, malnutrition, systemic diseases such as diabetes, etc. with delayed wound healing, altered levels of cytokines and inflammatory cells, decreased reepithelialization, and of course immunosuppression. Furthermore, chronic wounds, such as PU, usually present dysregulation of cytokines and growth factors. A critical step for treatments may be to target these factors. [51] Several studies [52,53] have reported that GH, PRL, and IGF-I have a direct influence on cells involved with immunity (high-affinity PRL and GH receptors have been observed on a number of these cells) and modulate humoral and cellular immune functions. 

Other anabolic agents, such as anabolic steroids (derivates of testosterone, i.e., oxandrolone), might be useful in promoting healing of PU. A comprehensive review of the literature found one trial in which oxandrolone administered orally was compared with a dose of placebo on pressure ulcer healing in people with spinal cord injuries [54]. The authors were uncertain whether oxandrolone is better than placebo in promoting complete healing of pressure ulcers at 24 weeks of treatment, and it could not draw conclusions about the potential benefits or harms of this treatment on treating PU. Well-designed studies are necessary to provide evidence as to whether anabolic steroids are beneficial or not in treating pressure ulcers.

We are concerned about the adverse effects of systemic GH treatment. Most side effects of GH treatment are local reactions at the injection site, such as pain, erythema, nodules, bruising, lipoatrophy, or swelling. However, different side-effects (i.e., hypoglycemia, changes in mental status, edema, fatigue, and headache) have been reported after systemic GH treatment and they are dependent on dose and time of administration. [26] Therefore, we developed our study for local administration of rhGH into ulcer edges. Several animal studies support the use of local GH in order to accelerate the wound healing process. Rasmussen et al. [55] injected different doses of GH into the back of 36 rats and compared it to a control group. They concluded that the optimal doses to increase the granulation tissue were between 0.2 to 0.7 UI. Kim et al. [56] demonstrated that the wound healing was faster after applying local rhGH in the back of five micro-pigs. A study by Lee et al. [22] described how the GH enhances the local formation of IGF-1, which activates fibroblast proliferation and keratinocyte migration—which highlight the potential of the topical application of GH. Andreassen et al. [32] reported bone formation without an increase in muscle mass, weight, or contralateral bone dimensions after the local injection of GH at the surface of tibial diaphyses in rats. Similar results were found in our study: mice did not present significant weight gain or changes in the usual behavior. Based on these data, we think that local application of GH should be the selected route of administration in future studies.

There is a lack of controlled clinical trials on humans to prove that GH can accelerate the PU healing process. There are a few case reports in the literature using the GH to treat PU. In 1955 Ravina et al. [57] reported the use of systemic GH to treat ulcers of diverse etiology. Six patients received intramuscular administration of the hormone. He reported complete healing after 1 to 3 months in a mixed cohort of patients with no control group and with different doses of GH. In 1987, Waago [58] administered GH topically in a diabetic patient with two recalcitrant ulcers. Four IU were administered topically twice per day, and 8 IU afterward. The author observed faster wound healing and noticeable decreased size of the ulcers. Due to logical ethical considerations, there was not histological analysis in all these cases to prove an improvement of the quality of the skin. The main advantages of our model are the possibility of having a matched control group and performing histological analysis without the previous ethical considerations.

As in most studies, this article has its limitations. A limitation of this study is the animal model. It is not clear how mouse cells could influence the ulcer healing process. Special care was taken to keep healthy human skin around the ulcer. The clip was placed in the center of the skin graft, maintaining healthy human skin around the ulcer, and injecting the rhGH into human skin, so that the healing and re-epithelialization were carried out by surrounding human cells (without the involvement of mouse skin). The human tissue over the mouse was assessed using fluorescence in situ hybridization (FISH) for chromosomes XX and XY, as previously described in *Maldonado* et al. [59]. We took advantage of the condition that the model was performed on male mice (XY chromosomes), and the human skin graft came from human female donors (chromosomes XX). Chromosomes XX were found in the areas of human skin graft. We think this model provides the opportunity to test different therapeutic strategies directly on human skin in the context of a living organism, without the ethical considerations involved in human research. A second bias is the initial pressure ulcer sizes: the control group presented smaller ulcers compared to the rhGH group. However, it is reported that wound contraction is 0.6–0.75 mm/day and keratinocyte migration up to 0.5 mm/day, regardless of the wound size [60], and therefore the initial size of the ulcer was not considered in the process of randomization. Finally, there are other histological techniques that could have provided useful information (i.e., the Fontana Masson Picrosirius technique [61] for identification of pigmented melanocytic lesions and the correlation between normal and neoplastic pigmented cells). Future studies will have to developed new observations and ideas. In spite of these limitations, we think our model opens up prospects for expanding knowledge about multiple fields such as skin wound healing, mechanisms of tolerance and immunological rejection, skin diseases, and carcinogenesis, among others. As we did in this study, cell therapy, growth factors, and other therapeutic strategies can be tested directly on damaged human skin through our ulcer model, without the ethical considerations involved in human research. Advances in skin healing and skin regeneration performed on our model could be potentially applied in clinical practice.

## 4. Materials and Methods 

### 4.1. Animals

Three-week-old, male, non-obese diabetic/severe combined immunodeficiency (NOD/Scid-NOD.CB17-Prkdscid/NCrHsd) mice (*n* = 10) (Harlan Laboratories S.r.l. Barcelona, Spain) were used in this study. These mice present immunological multidysfunction, including absence of mature T and B cells, reductions in macrophage function, complement-dependent hemolytic activity, and NK cell activity. [62] All mice were caged under standard light and temperature conditions with free access to food and water throughout the study. All efforts were made to minimize suffering and all animals were sacrificed at the end of the study.

All experimental procedures were approved and regulated by the Animal Experimentation Ethics Committee—University of Alcalá and University Hospital of Getafe (IRB: PROEX reference 237/15, 9 October, 2015), in accordance with the Royal Decree 53/2013 and the European Community Council Directive. 

### 4.2. PU model on Human Skin

Ten NOD/Scid mice were engrafted under general anesthesia (Ohmeda, BOC Health Care) with female human skin. All human skin (*n* = 5) came from abdominoplasty procedures. Written informed consent was obtained from all patients. The skin was immediately stored after haverst in D-MEM (Dulbecco/Vogt modified Eagle’s minimal essential medium) at a temperature of 4 °C. Skin grafting was performed during the first 24 h after extraction. Human full-thickness skin grafts (FTSGs) were placed onto a 4 × 3 cm wound. Mice skin was incised down to the muscle and removed, exposing muscular layer. FTSGs were sutured in place with 4/0 nylon. A tie-over bolster dressing was placed for the first 5 days. The same skin donor was used to graft two mice (in order to pair two mice with the same human skin donor). After 60 days, a compression device was applied to the human skin graft, as previously described. [59] The clip exerted a pressure of 150 mmHg, measured with a dynamometer. Three cycles of compression–release (8 h of clamping after 16 h of no compression) were applied to simulate a pressure ulcer, based on the method described by Stadler et al. [63].

### 4.3. Recombinant Human Growth Hormone (rhGH)

Genotonorm Kabipen 5.3 mg (Pfizer, Madrid, Spain), solution for injection in a pre-filled pen, was provided by the Department of Pharmacy of the University Hospital of Getafe and stored at a temperature between 2 and 8 °C. The use of the rhGH is restricted to the hospital setting, and its use was approved and regulated by the Animal Experimentation Ethics Committee University Hospital of Getafe (PROEX reference 237/15) in accordance with the Royal Decree 53/2013 and the European Community Council Directive. 

### 4.4. Local Treatment of the PU with rhGH

**Figure 8:**
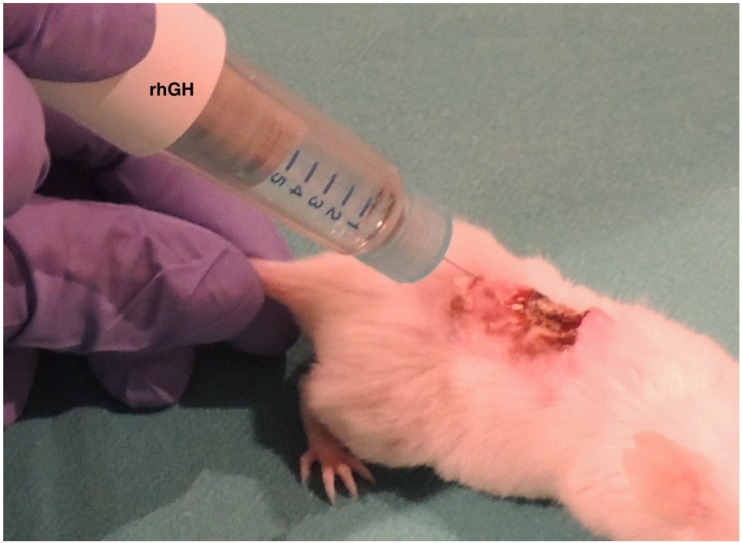
Local administration of the rhGH. Note the intradermal injection of the rhGH applied to the pressure ulcer edges on human skin.

PU formation was confirmed by visual assessment after the last cycle of compression–release. Paired mice were assigned randomly to the rhGH group (*n* = 5) or to the control group (*n* = 5). We designed a protocol for local administration of the hormone. The concentration of GH was determined based on previous studies. [55] In the rhGH group, the five mice were treated with four local rhGH intradermal injections, each of 0.15 mg (0.5 IU), applied to the PU edges on human skin (Figure 8). The injection protocol started 24 h after the last cycle of compression–release, and the administration of the hormone was repeated once per week for four weeks, always the same day of the week at the same hour. In the control group, the five mice received the same care but without the local administration of the rhGH.

### 4.5. Macroscopic Analysis

Evaluations were conducted with photographic and visual assessment in both groups. PU size (in mm^2^) was measured every seven days until complete healing (wound closure) using the software ImageJ (National Institute of Health, USA). The healing rate was calculated in both groups as the initial size of the ulcer in mm^2^ per number of days until closure. The weight in grams of all mice was recorded before creating PU, after the three cycles of ischemia-reperfusion, and once per week until wound closure. All the measurements were taken by two independent researchers.

### 4.6. Microscopic Evaluation

Histological analysis of the human skin was performed in both groups after wound closure by two independent histologists. Samples of skin were obtained from the two groups, and they were placed in 10% buffered formaldehyde (for histopathological studies) and Bouin (for immunohistochemical studies). Then the samples were dehydrated and embedded in paraffin. Tissue sections (5 mm thick; 50 sections for each sample) passing through the central plane of each sample were stained with hematoxylin-eosin and Masson’s trichrome for morphological assessment. Tissue sections were examined under a Zeiss Axiophot light microscope (Carl Zeiss, Oberkochen, Germany). The thickness of the healed human skin was measured in micrometers with Axiovision Release 4.6.3 software (Carl Zeiss, Oberkochen, Germany). The total thickness of the skin was defined as the distance between the stratum corneum and the boundary between human dermis–host mouse tissue. 

### 4.7. Immunohistochemistry

Samples of tissue were deparaffined, hydrated, and equilibrated in phosphate buffered saline (pH 7.4). Rabbit polyclonal anti-COL-I and anti-COL-III (Abcam, Cambridge, UK) were used as primary antibodies to study collagen type I and III. Samples were incubated with secondary antibodies, anti-rabbit immunoglobulin G (IgG) (Sigma-Aldrich, St. Louis, MO, USA). For COL-I and COL-III, the alkaline phosphatase procedure was performed. COL-I and COL-III were conjugated with avidin-alkaline phosphatase (ExtrAvidin-Alkaline Phosphatase, Sigma-Aldrich, St. Louis, MO, USA). It was used for 60 min at room temperature (Dilution 1/200 in PBS) and developed with the alkaline chromogenic substrate for 15 min (controlling the appearance of marking under the microscope). The chromogenic substrate preparation was performed immediately before development by adding 10 mL of PBS (10 mg of α-naphthol AS-BI phosphate, 10 mg of Fast Red, and 100 μL of 0.1 M levamisole) [64]. Nuclei were counterstained with Carazzi’s hematoxylin (15 min). A negative control of the technique was performed without the primary antibody. Samples were mounting in aqueous medium with Plasdone. Tissue sections were examined under a Zeiss Axiophot light microscope equipped with an AxioCam HRc (Carl Zeiss, Oberkochen, Germany). A digital camera was used for observations. In order to calculate the percentage of protein expression, the German semiquantitative scoring system considering the staining intensity and area extent was used [65]. Immunostaining in the tissue was assessed by two independent histologists. 

### 4.8. Statistical Analysis

The results were analyzed by the software SPSS 21 (SPSS, Inc., Chicago, IL, USA). Sample size estimation was performed considering a statistical power of 0.8 and alpha error of 0.05. Mean values of healing rates, thickness of the skin and collagen type I and III expression were compared between groups using Mann-Whitney’s U test. Differences with *p*-value < 0.05 were considered statistically significant.

## 5. Conclusions

Based on our model, local administration of the rhGH accelerates the wound healing process and improves the quality of human skin. The healing rate was twice as fast in the rhGH group compared to the control group. In the rhGH treatment group, we found an increase in dermis regeneration tissue with a delay in the maturation period, which potentially improves the re-epithelialization process. Based on the macroscopical and histological findings and considering the human skin component of this model, the rhGH may have a clinical use in pressure ulcer treatment, and further studies remain to be performed. We think that our findings could be extrapolated to other compromised wound healing situations such as diabetic or chronic ischemia patients. 

## Figures and Tables

**Table 1:** Percentage of protein expression (collagen type I and III) in the total area of tissue samples (the rhGH(recombinant human growth hormone) group and the control group).

	Collagen type I	Collagen type III
rhGH group	16.8 % ± 4.4	70.8 % ± 2.8
Control group	76.8 % ± 16.5	64.4 % ± 3.8
*p*-value	<0.01	<0.05
